# Use of principle component analysis to quantitatively score the equine metabolic syndrome phenotype in an Arabian horse population

**DOI:** 10.1371/journal.pone.0200583

**Published:** 2018-07-12

**Authors:** Samantha L. Lewis, Heather M. Holl, Maureen T. Long, Martha F. Mallicote, Samantha A. Brooks

**Affiliations:** 1 Department of Animal Sciences, University of Florida, Gainesville, FL, United States of America; 2 Department of Infectious Diseases and Pathology, College of Veterinary Medicine, University of Florida, Gainesville, FL, United States of America; 3 Department of Large Animal Clinical Sciences, College of Veterinary Medicine, University of Florida, Gainesville, FL, United States of America; University of Missouri Columbia, UNITED STATES

## Abstract

Equine metabolic syndrome (EMS), like human metabolic syndrome, comprises a collection of clinical signs related to obesity, insulin dysregulation and susceptibility to secondary inflammatory disease. Although the secondary conditions resulting from EMS can be life-threatening, diagnosis is not straightforward and often complicated by the presence of other concurrent conditions like pituitary pars intermedia dysfunction (PPID). In order to better characterize EMS, we sought to describe the variation within, and correlations between, typical physical and endocrine parameters for EMS. Utilizing an unsupervised statistical approach, we evaluated a population of Arabian horses using a physical examination including body measurements, as well as blood plasma insulin, leptin, ACTH, glucose, and lipid values. We investigated the relationships among these variables using principle component analysis (PCA), hierarchical clustering, and linear regression. Owner-assigned assessments of body condition were one full score (on a nine-point scale) lower than scores assigned by researchers, indicating differing perception of healthy equine body weight. Rotated PCA defined two factor scores explaining a total of 46.3% of variation within the dataset. Hierarchical clustering using these two factors revealed three groups corresponding well to traditional diagnostic categories of “Healthy”, “PPID-suspect”, and “EMS-suspect” based on the characteristics of each group. Proxies estimating up to 93.4% of the composite “EMS-suspect” and “PPID-suspect” scores were created using a reduced set of commonly used diagnostic variables, to facilitate application of these quantitative scores to horses of the Arabian breed in the field. Use of breed-specific, comprehensive physical and endocrinological variables combined in a single quantitative score may improve detection of horses at-risk for developing EMS, particularly in those lacking severe clinical signs. Quantification of EMS without the use of predetermined reference ranges provides an advantageous approach for future studies utilizing genomic or metabolomics approaches to improve understanding of the etiology behind this troubling condition.

## Introduction

Equine Metabolic Syndrome (EMS) is a condition characterized by regional and abnormal adiposity, hyperinsulinemia, and susceptibility to laminitis [1, 2]. Characteristics of the EMS phenotype, particularly in early stages, can be difficult to identify; often requiring endocrine diagnostics like the dynamic test for insulin dysregulation [3, 4]. Since limited treatments are available for EMS, diagnosis and prevention are of utmost importance [2, 4]. Current recommendations for EMS emphasize management of obesity through careful diet and exercise regimens [5, 6]. Overlap of clinical signs for Pituitary Pars Intermedia Dysfunction (PPID), also known as Equine Cushing’s disease, creates additional difficulty in diagnosis [7]. Affecting primarily older horses, PPID is the result of dopaminergic neurodegeneration of the pars intermedia of the pituitary gland. Clinical signs include hypertrichosis, polyuria, abnormal adiposity, and laminitis, which in some PPID horses may be due to endocrinopathic insulin dysregulation [3, 8]. PPID can exist concurrently with EMS, and there is some evidence suggesting that underlying EMS may be the cause of overlapping characteristics of these two conditions, like insulin dysregulation and laminitis, as these findings are not present in all PPID cases [3, 8, 9].

Characterized as a cluster of signs associated with laminitis susceptibility, EMS may present physiologically as obesity, insulin dysregulation and hyperlipemia [10]. In this definition, obesity is the only outwardly observable clinical sign. In many cases obesity is present with insulin dysregulation, yet regional obesity may be absent in some horses with EMS [2, 11]. The Henneke body condition scoring (BCS) system is widely used to classify obese or overweight horses, though it is at best only semi-objective [12]. Skillfully applied, the BCS is an accurate estimate of obesity, yet few owners receive specific training or practice in its use and, as a result, commonly underestimate body condition of their horses [13]. Morphometric measurements including the neck circumference at midpoint and heart girth circumference, relative to the height at the withers (NC/H and HG/H), can provide a quantitative estimate of obesity [14, 15]. These measures of obesity may inform predictive estimates of future laminitis risk and/or a “pre-laminitic” condition, but the most informative measure may differ by breed and/or the tendencies of the individual horse [16, 17].

Insulin dysregulation, stemming from excessive insulin secretion and peripheral insulin resistance, is the main endocrinopathy proposed in EMS [10]. Further tests such as circulating triglycerides, cholesterol, and leptin concentrations may be useful as elevated values are indicative of EMS [18, 19]. Additional recommendations include diagnostic testing to rule out PPID as a possible diagnosis [20, 21]. Although functional tests for insulin resistance and pituitary function are preferable, ambulatory veterinarians in the field may be limited to one-time basal glucose and insulin tests and circulating ACTH levels due to various constraints including cost to owner, access to a laboratory for rapid processing, and limited time [10]. Two previously published proxy measurements improved estimates of IR using a single basal glucose and/or insulin concentration [22]. The reciprocal of the square root of insulin (RISQI) estimates insulin sensitivity of peripheral tissues (RISQI) and the modified insulin-to-glucose ratio (MIRG) estimates β-cell response to glucose [22].

Unsupervised modeling methods utilizing multiple easily measurable physiological variables, including body measurements and serum diagnostics, might present a solution to increase accuracy when screening for EMS. In this type of approach, variable reduction methods like Principle Components Analysis (PCA) are commonly applied to complex diagnostic datasets for conditions as diverse as altitude sickness, Schizophrenia, and most relevant here, human Metabolic Syndrome [23–25]. PCA efficiently condenses these multivariate datasets in to a single vector explaining the largest possible proportion of variation within the dataset, and does not require *a priori* knowledge of “gold-standard” measures or underlying variables. The main goal of this study was to couple quantitative body scoring measures with single time point tests in order to describe and refine field-based testing for EMS. To this aim, we used principle component analysis (PCA) and hierarchical clustering to transform nine physiological variables into a comprehensive score for characteristics of EMS within the Arabian horse population. We then determined prediction expressions to simplify future investigations comparing these analyses against functional testing, longitudinally within our study cohort, and for evaluation of this approach across multiple breeds. Ultimately, this work will contribute to improved field screening methods for identifying horses with EMS.

## Materials and methods

### Study protocol

All work was approved by University of Florida Institutional Animal Care and Use Committee and carried out by researchers accordingly (protocol #201408459). Owners of registered purebred Arabian horses were recruited through email, referring veterinarians, and online advertisements. Written consent was attained from the owner for each horse participating in this study.

### Sample collection

Horses were initially selected for participation in the study based on responses from an owner-completed online survey comprising 73 questions collected using Qualtrics survey software (Qualtrics, Provo, Utah). Surveys provided background information for each horse including descriptions of diet and exercise, age, sex, registration number, and owner assigned BCS. Additional medical history included previous diagnosis of disease, particularly of EMS or PPID, current medications, and any history of laminitis. Out of 109 horses volunteered through the initial online survey, 50 were excluded due to insufficient age (target age range >8 years old), or were currently receiving medications that could interfere with test results (primarily phenylbutazone in the case of the lameness exam, as well as cyproheptadine, and pergolide). Horses used in this study were also utilized in a concurrent genome-wide association, therefore [26], this effort targeted only pure-bred Arabians and some candidates were excluded due to mixed or unknown ancestry. Privately-owned farms were visited for sampling if at least one horse present was previously diagnosed or suspected, by a veterinarian or owner, of suffering from EMS. As many additional horses as possible, matching the study criteria for breed, sex, and age, were also sampled at each participating farm. Overall, 73 horses were enrolled, with nine horses excluded post-collection due to poor owner compliance with pre-testing fasting requirements, inability to verify purebred Arabian ancestry, or incomplete owner surveys. The final population dataset consisted of 34 non-pregnant females and 30 castrated males, aging from 8–34 years old with a mean of 16.45 years.

Samples were collected at 19 farms across north central Florida, from August to November 2014. Horses were housed indoors or at pasture and participated in various activities including breeding, pleasure, and performance (dressage, hunter, and endurance). Farm visits occurred between 6am and 10am, and owners were instructed to withhold feeding of any concentrated rations for 12 hours prior to the examination, though horses were allowed continual access to forage. Body measurements collected from each horse included heart girth (HG), height at withers (H), and neck circumference (NC) at mid-point from poll to withers, using measuring tape at landmarks previously established [14]. BCS assessed independently by three members of the research team was averaged for analysis (AVG BCS). During examinations, veterinarians recorded heart rate and respiration rate as part of an overall assessment of health. Furthermore, pasture asthma and anhidrosis are common conditions of mature horses in central Florida, warranting use of heart rate and respiration rate as sentinel diagnostics to catch suspected cases of these conditions.

Blood samples were collected by jugular venipuncture in two 10 mL EDTA Vacutainer vials (BD Vacutainer®, Becton, Dickinson and Company, Franklin Lakes, NJ) for diagnostic endocrinology and a six mL potassium oxalate Vacutainer vial (BD Vacutainer®, Becton, Dickinson and Company, Franklin Lakes, NJ) for glucose concentrations. On-farm processing of potassium oxalate and EDTA vials consisted of centrifugation at 500 *g* for 10 minutes, transfer of plasma by pipette to 1–2 mL aliquots into cryovials and flash freezing by immersion in liquid nitrogen. Aliquots were moved for storage to a -80°C freezer within four hours of collection. Freezing of plasma samples in liquid nitrogen directly after collection minimalized variation in sample quality due to inconsistent handling, environmental conditions and degradation. Potassium oxalate preserved aliquots of plasma were submitted to University of Florida Veterinary Diagnostic Research Laboratory for measurement of glucose concentrations (mg/dL) using the Dimension® Xpand Plus integrated chemistry system (Siemens, Erlangen, Germany). EDTA preserved aliquots of plasma were submitted to the Cornell Animal Health Diagnostic Center (accredited by American Association of Veterinary Laboratory Diagnosticians and USDA) for ACTH, leptin, cholesterol and triglycerides. Cholesterol and triglycerides were measured using a Roche ModP chemical analyzer (Roche Diagnostics Indianapolis, Indiana), leptin was measured using the Millipore Multispecies Leptin RIA (Linco Research Inc., St. Louis, MO), and insulin was measured using Millipore porcine insulin RIA kit (EMD Millipore Corporation, Darmstadt, Germany) as previously described[27]. The ACTH measurements were performed using an automated chemiluminescent enzyme immunoassay system (Immulite, Diagnostic Products Corporation, Los Angeles, CA), previously validated by Perkins *et al*., 2002 [28].

Relative levels of exercise, as reported in owner surveys, were determined using published parameters [29]. Ambient temperature was established based an average morning temperature from online records (www.wunderground.com) according to date and location.

Body measurements for each horse were calculated into ratios HG/H and NC/H, [14] as obesity estimates for comparison to BCS. Insulin dysregulation proxies were calculated using basal insulin and glucose concentrations including glucose:insulin ratio, reciprocal inverse square of basal insulin (RISQI), and modified insulin-to-glucose ratio (MIRG) [22].

$$RISQI=\frac{1}{\sqrt{insulin}}$$

$$MIRG=\frac{800-0.30\;{\left[insulin-50\right]}^{2}}{glucose-30}$$

### Statistical analyses

Values for age, NC/H, HG/H, AVG BCS, ACTH, leptin, MIRG, triglycerides, and cholesterol were analyzed by Principle Component Analysis (PCA). Shapiro-Wilk tests determined a non-normal distribution for variables of age, plasma ACTH and plasma leptin. Therefore, a Varimax factor rotation was performed on the two principle components determined to be statistically significant by the Bartlett test of eigenvalues (p < 0.05). Samples were clustered using a hierarchical approach into three groups based on factors 1 and 2 (assuming three possible diagnoses). To illustrate trends between the resulting clusters and the original variables, ANOVA tests were used to assess seven of the original nine variables. Two variables, ACTH (log transformed for normalcy) and triglycerides, failed a Levene’s test for unequal variances and were therefore described using a Kruskal-Wallis test. A standard least square model estimated the original two factor scores using a reduced set of diagnostic measures that were more typical of tests performed in a clinical setting. Remaining measures not used in the PCA were including vital signs, exercise level and ambient temperature, were evaluated for relationships using ANOVA tests. All statistical analysis was performed using JMP 12® (SAS Institute, Cary, NC) [30].

## Results

**Fig 1:**
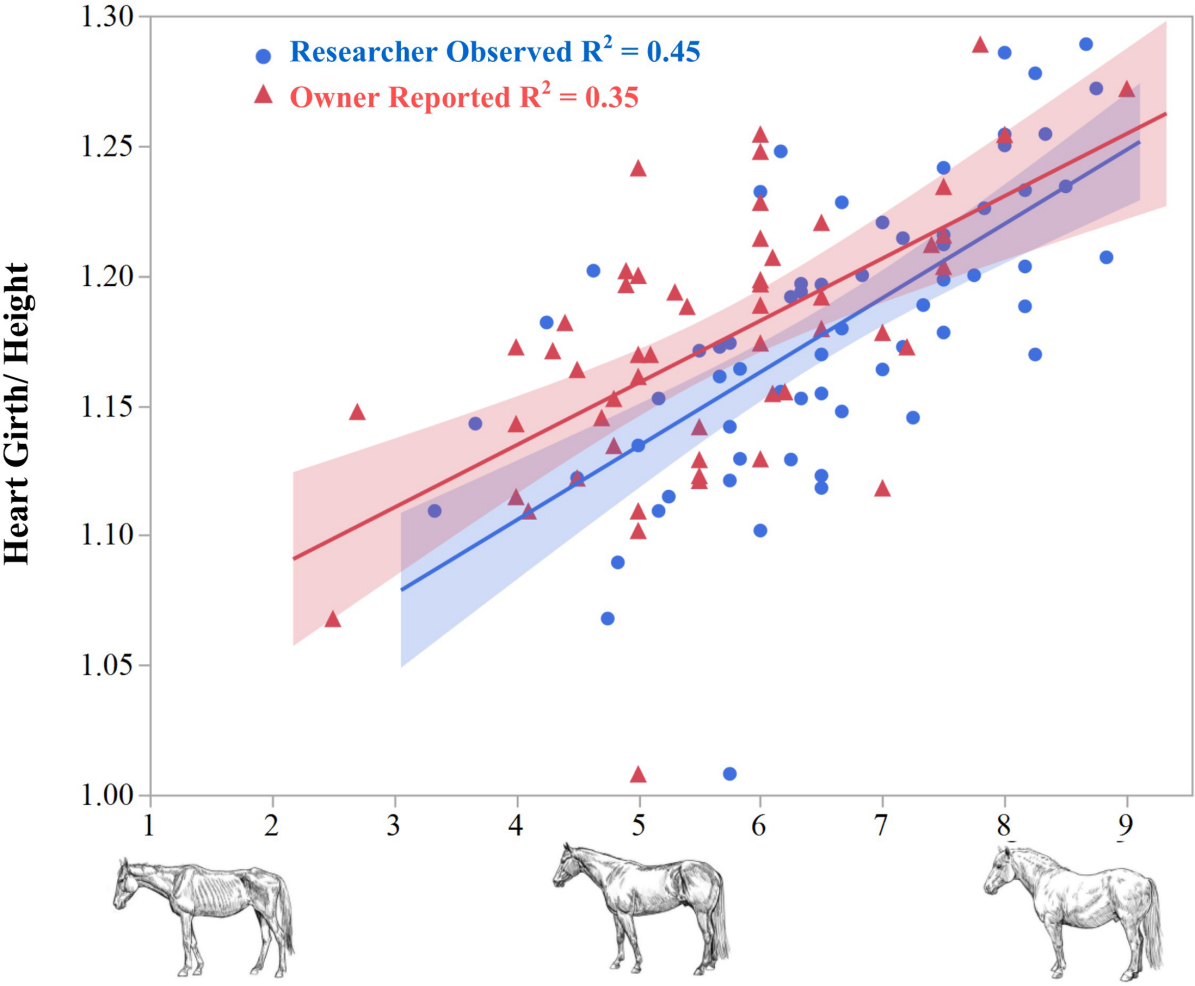
Owner versus researcher reported BCS compared to the heart girth/height ratio. Owner reported body condition scores (BCS, triangles) underestimated obesity in their horse when compared to the researcher observed values (circles), potentially contributing to overfeeding. Both BCS observations correlated well with a ratio of heart girth circumference to height, suggesting the objective measurement method was an adequate proxy for adiposity/BCS.

Owner reported BCS values underestimated the adiposity of their animals by an average of 1.0 BCS unit (p < 0.0001) compared to the researcher average scores (Fig 1). The ratio of heart girth to height (HG/H) correlated positively with both owner-reported and averaged researcher BCS scores. This indicates that owner underestimation of adiposity in their horses occurred almost uniformly across the range of the BCS scale, and demonstrates the utility of body measures as a quantitative estimation for obesity.

As expected, resting heart and respiration rate on the day of sampling did not correlate with endocrine values. Heart rate positively correlated with AVG BCS (R^2^ = 0.074, p = 0.033) and inversely with exercise level (R^2^ = 0.24, p = 0.0024). Respiration rate positively correlated with the presence of a sweat dampened hair coat at the time of sampling (R^2^ = 0.19, p = 0.0003) and with ambient temperature (R^2^ = 0.22, p < 0.0001). BCS was not related to owner-reported exercise level (p = 0.80) but was inversely correlated with age (R^2^ = 0.12, p = 0.0045). HG/H was the only body measurement to significantly differ by sex, as females had higher HG/H than castrated males (R^2^ = 0.14, p = 0.0011). In a comparing obesity measurements with previously described insulin dysregulation estimators, only MIRG and RISQI were significantly correlated to all three morphometric measures, HG/H, NC/H and AVG BCS scores (Table 1).

**Table 1:** Correlations of obesity measures to various insulin and glucose ratios (R^2^, P-values by ANOVA).

Measure (R^2^, P-values)	HG/H	NC/H	BCS
Insulin (FSIT)	0.059, 0.052	0.075, 0.028	0.087, 0.018
RISQI	0.067, 0.040	0.074, 0.030	0.068, 0.037
MIRG	0.089, 0.017	0.076, 0.028	0.078, 0.026
Glucose: insulin	0.069, 0.037	0.065, 0.041	0.053, 0.067

**Fig 2:**
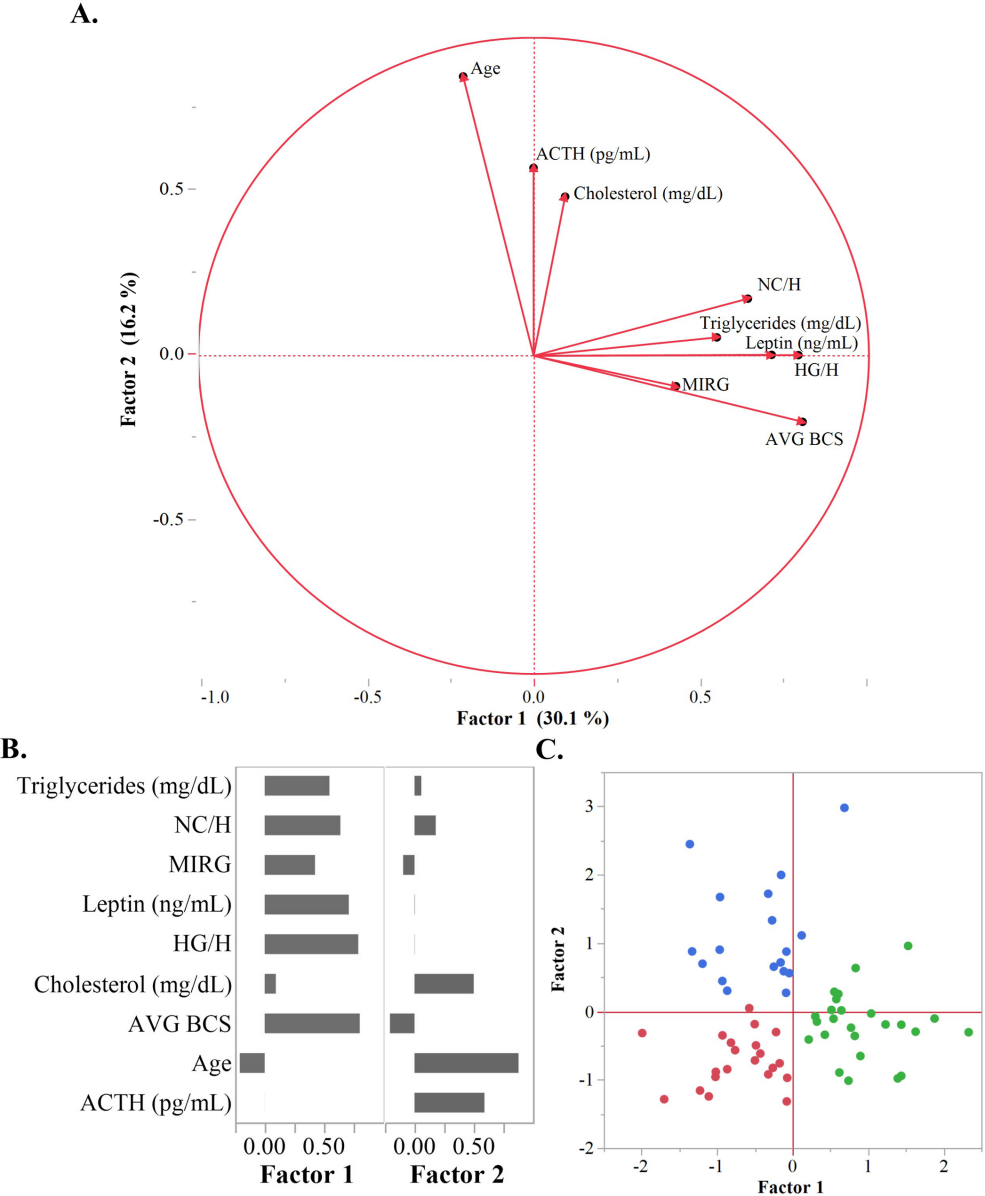
Principle component analysis for EMS phenotype. (A) Vector loading plot of Factor 1 vs Factor 2, Factor 1 characterizes the EMS-suspect phenotype and Factor 2 the PPID-suspect phenotype. (B) Eigenvectors for each factor plotted against the original nine diagnostic variables. Factor 1 positively correlates with Triglycerides, leptin, MIRG, BCS, NC/H, and HG/H while Factor 2 correlates with age, ACTH, cholesterol and NC/H. (C) Factor 1 and Factor 2 scores plotted for each horse and colored according to hierarchical cluster: healthy (red), PPID-suspect (blue), and EMS-suspect (green).

Correlative PCA followed by factor rotation on nine physiological measures distilled relationships between these variables into two factor scores, Factor 1 and Factor 2, explaining 30.1% and 16.3% of the variation respectively (Fig 2). Factor 1 correlated positively with variables typically elevated in obesity and EMS, including AVG BCS, NC/H, HG/Height, triglycerides, leptin, and MIRG. Factor 2 positively correlated PPID characteristics; age, cholesterol, and ACTH.

**Fig 3:**
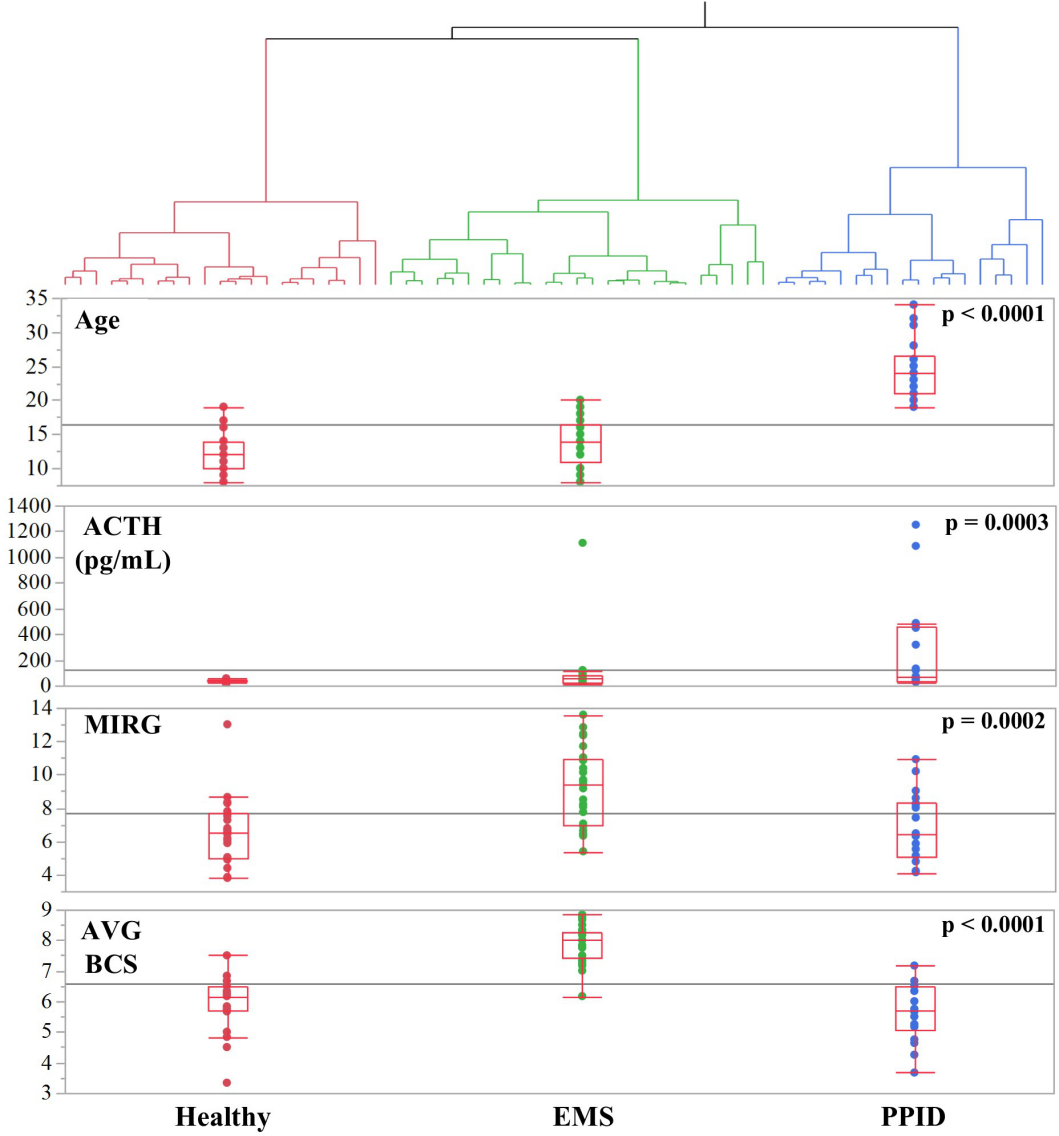
Clusters based on PCA factors. Hierarchical clustering of horses into three diagnostic categories, Healthy (red), EMS-suspect (green), and PPID-suspect (blue), using Factor 1 and Factor 2. Below clusters, four of the nine variables from the PCA across disease clusters displaying quartiles (top and bottom of red box) and median (middle line across box) using whisker-bow plots. All p-values calculated by ANOVA tests. The PPID-suspect cluster had the highest Age (p < 0.0001) and ACTH (p = 0.0003). The EMS-suspect cluster had the highest AVG BCS (p < 0.0001) and MIRG values (p = 0.0002).

Hierarchical clustering divided the study population into three groups based on the factor 1 and factor 2 scores for each animal (Fig 3). These clusters were termed the “Healthy” (n = 21), “PPID-suspect” (n = 28), and “EMS-suspect” (n = 25) groups based on the distributions of the original nine variables within each group (Table 2). The “PPID-suspect” group had significantly higher cholesterol, age and ACTH concentrations, compared to the “EMS-suspect” and “Healthy” clusters. AVG BCS, HG/H, NC/H, leptin, and triglycerides were significantly higher in the “EMS-suspect” group compared to the others.

**Table 2:** Summary statistics (mean ±SD) for diagnostic measures by score-defined clusters.

Factor	Variable	Healthy	PPID-suspect	EMS-suspect	Raw P^‡^
**1**	AVG BCS	5.91 ±0.91^a^	5.64 ±0.94 ^a^	7.82 ±0.63 ^b^	<0.0001
**1**	HG/H	1.14 ±0.042 ^a^	1.16 ±0.044 ^a^	1.22 ±0.038 ^b^	<0.0001
**1**	NC/H	0.58 ±0.028 ^a^	0.59 ±0.034 ^a^	0.62 ±0.033 ^b^	0.0002
**1**	MIRG	6.61 ±2.04 ^a^	6.83 ±2.09 ^b^	9.21 ±2.31 ^a^	0.0002
**1**	Leptin (ng/mL)	3.06 ±1.53 ^a^	4.12 ±1.55 ^a^	7.81 ±4.07 ^b^	<0.0001*
**1**	Triglycerides (mg/dL)	20 ±8.67 ^a^	27 ±12.60 ^b^	39 ±15.41^c^	<0.0001^**†**^
**2**	ACTH (pg/mL)	37.3 ±10.83 ^a^	266.5 ±366.47 ^c^	94.4 ±213.37 ^b^	0.0026*^†^
**2**	Age (years)	12 ±2.83^a^	25 ±4.22^b^	14 ±3.44^a^	<0.0001*
**2**	Cholesterol (mg/dL)	83 ±13.2 ^a^	95 ±14.5 ^b^	86 ±9.7 ^a^	0.0108
**-**	Insulin (FSIT, μIU/mL)	14.5 ±7.47	13.9 ±6.57	24.4 ±1.63	<0.0001
**-**	Glucose (mg/dL)	91.6 ±6.78	88.3 ±6.99	93.1 ±1.34	0.0757
	**Factor 1 score**	-0.716 ±0.513 ^a^	-0.460 ±0.569 ^a^	0.933 ±0.541 ^b^	<0.0001
	**Factor 2 score**	-0.722 ±0.378 ^a^	1.12 ±0.761 ^c^	-0.199 ±0.475 ^b^	<0.0001

^‡^ ANOVA, unless otherwise noted

* variable log-transformed

^†^ raw p-values based on Kruskal-Wallis

^a, b, c^ indicators of significant difference between clusters

Linear regression of variables on PCA factors provided reduced models for estimating the factor scores using a reduced set of diagnostics as follows:

$$Predicted"EMS-suspect"score=-16.34+(0.084\;x\;MIRG)+(13.30\;x\;\frac{HG}{H})$$

$$Predicted"\mathrm{EMS}‑\mathrm{suspect}"score\;(with\;Leptin)=-13.22+(0.004\;x\;MIRG)+(10.25\;x\frac{HG}{H})+(0.545\;x\;LogLeptin)$$

Predicted"PPID−suspect"=−6.05+(1.88xLogAge)+(0.22xLogACTH)

Only 77.6% of variance in the original Factor 1 “EMS-suspect” score was captured by a reduced model utilizing only the MIRG and HG/H measures (compared to only 21.2% explained by MIRG alone). With the addition of plasma leptin values to MIRG and HG/H, the prediction equation captures 93.4% of the variance of the full model. Similarly, a combination of age and ACTH explained 99.9% of variance in Factor 2, versus 95% with ACTH alone.

## Discussion

The overall goal of this study was to use unsupervised statistical modelling to describe the variation within the obesity and EMS phenotype in an Arabian horse population, without the constraint of a prior diagnosis. The variables utilized consisted of relatively straightforward body measurements and scores, combined with commonly used clinicopathological indices of insulin, glucose, lipids, leptin, and ACTH. Application of an unsupervised statistical method allowed examination of variation among our diagnostic criteria without assumptions derived from previously published “gold-standard” references ranges and disease definitions. The factor scores illustrate the spectrum-like range of severity for this condition, allowing independent placement across the “PPID-suspect” or “EMS-suspect” axes with varying magnitude. This quantitative approach more closely resembles the spectrum of clinical signs observed in these conditions than neat diagnostic categories based on a single assay.

The utilization of a numerical method for quantifying obesity was necessary in order to perform PCA analysis. This is an important point in our study, as only numerical values are suitable for PCA. Owners consistently underestimated the body condition of their horses. Owner provided scores were nearly one unit lower than the researchers averaged scores across the entire breadth of the scale, supporting previous findings [13, 31]. This disagreement may stem from insufficient training in use of the BCS system, or from a human tendency to be overly-optimistic in diagnosis of obesity in companion animals [32]. Our findings support the HG/H ratio as a quantitative measure for obesity as it correlated well with BCS and “EMS-suspect” score, in agreement with previous findings [15, 33]. We observed significantly higher HG/H measures in non-pregnant females than in castrated males. This could be result of a higher prevalence of EMS among females, or a physiological tendency for mares to deposit more adipose tissue around the heart girth area.

Although HG/H alone is not sufficient for estimation of body weight, it may be an ideal measurement for estimation of adiposity by owners as they can repeatedly find these landmarks on their horse and the procedure is similar to the commonly used calibrated “weight tapes” [34], The heart girth measurement alone reveals more subtle changes in adiposity compared to BCS. [35, 36]. Allowing owners to more accurately measure small body mass changes could result in increased owner compliance to stricter feeding regimens and earlier prediction of weight gain and inappropriate deposition of fat.

Factor 1 (“EMS-suspect” score) positively correlated with variables typically characteristic of EMS including obesity measurements, MIRG, leptin, and triglycerides [15, 18]. One aspect of this study was the lack of severe EMS cases within our population. Severe hyperinsulinemia (circulating insulin above 70 μIU/mL) and/or a history of severe laminitis did not occur in within our sample set [37]. This may be a result of the participant screening process, in which horses administered medications were excluded. Horses suffering from severe cases of EMS would likely be treated for laminitis or hyperinsulinemia with medications that would alter the physical exam, and consequently, confound our results. Only eight horses within our population could be traditionally deemed EMS cases as they exceeded all three published screening guidelines for EMS diagnosis including a BCS of seven or greater, plasma leptin higher than seven ng/mL, and fasting insulin concentration over 20 μIU/mL [4]. Our EMS samples represented mild cases or early-stage EMS, also referred to as pre-laminitic metabolic syndrome, thus allowing specific examination of the early stages of the condition and those as risk for developing EMS. According to a previous report, elevated triglycerides and BCS function as proxies to indicate a pre-laminitic metabolic syndrome diagnosis in ponies [19]. Another study conducted in ponies suggested measurements of blood pressure and dynamic measures of insulin dysregulation as useful in early detection of EMS [38]. Within our population of Arabian horses, the “EMS-suspect” score correlated with elevation in all three of these pre-EMS biomarkers, further supporting this summary score as an indicator of metabolic irregularities.

In attempt to lower costs accrued by multiple diagnostic tests, we were able to create a proxy estimating 77.6% of the variance captured by the “EMS” score using tests most frequently used for endocrine testing including fasting insulin and glucose, with the addition of HG/H. This proxy may be a more practical method for clinical application as it requires only one trip to the farm, and reduces cost for owners in comparison to the oral glucose tolerance test, combined glucose-insulin tolerance test, or a full diagnostic panel including leptin and triglycerides. However, the addition of leptin values to this model captures 93.4% of the variance of the “EMS-suspect” score, supporting the inclusion of leptin testing for EMS diagnosis.

We did not expect a significant amount of variance within our sample set to consist of characteristics of a condition that was specifically excluded prior to collection. Our ACTH values were recorded during late summer/fall when ACTH is normally elevated, and the magnitude of this elevation is up to 2.8 times greater in PPID animals than those without [39, 40]. By collecting during late summer and fall, horses with underlying PPID that lacked visible signs of the condition were more easily identified than in other seasons, thus allowing for a more definitive distinction of PPID cases from EMS cases [40]. Yet, Factor 2 still captured significant variation in plasma ACTH and age, both key characteristics of PPID, suggesting the label “PPID-suspect” score. We identified many horses with severely elevated circulating ACTH concentrations that lacked clinical signs of PPID. These horses may have subclinical disease, be at risk of developing PPID in the future, or are simply exhibiting asymptomatic elevated ACTH concentrations due to old age or other conditions [21].

High circulating cholesterol is reported as a sign of EMS [41], yet we observed elevated cholesterol strongly correlated only to the “PPID-suspect” score, a finding more closely in line with those of Elzinga *et al*., 2016 [27]. The correlation of circulating cholesterol with the “PPID-suspect” score may result from endocrine dysfunction caused by PPID, or simply be a result older age. However, we observed that age and basal ACTH concentrations alone were sufficient to identify horses as possible PPID cases, as both variables explained 99.9% of variation in the original “PPID-suspect” score [21].

Heart and respiration rates reflected ambient temperature (perhaps a result of heat stress) not EMS or obesity. Fitness (as determined by owner-reported exercise activity) correlated with a lower resting heart rate among horses performing more intense exercise regimes. However, horses with lower BCS scores exhibited higher resting heart rates than their higher BCS counterparts. Therefore, vital signs and fitness were not reliable indicators of obesity or the EMS phenotype. Early identification of at risk individuals through other approaches offers opportunity for intervention such as weight loss and diet restriction, especially during spring and summer months to reduce the risk of developing laminitis [19].

Our results suggest consideration of EMS, especially in the early stages, as a complex condition that presents as a spectrum of disease, rather than a threshold diagnosis. This approach assessed the multidimensionality of EMS, allowing improved identification of horses with only mild EMS characteristics that could be at risk for developing clinical EMS. Proxies created in this study provide a reduced model for identifying horses at risk for EMS without added cost and labor of the full-scale model. Findings from this study may be useful to improve the accuracy of identifying individuals at-risk for EMS, particularly in the Arabian breed.

Longitudinal follow-up with the horses from this study may validate the predictive value of our scoring system. It should be noted that this study was performed on mature horses within a single breed displaying relatively mild clinical signs. Longitudinal studies are needed in broader populations of horses, including those with more severe EMS and/or PPID conditions, in order to evaluate the accuracy of our disease proxies. Evaluation of similar proxies in other breeds at risk for endocrine issues, such as Morgan horses, and in breeds with few EMS cases like the Thoroughbred, may be particularly relevant given suspected genetic predilections to this condition.

## Supporting information

S1 Table Raw diagnostic measures, derived normalized measures, resulting factor scores and clusters for the sampled population of horses. (XLSX)
